# Visions of the electrochemical future, past and present: Plus ca change?

**DOI:** 10.1007/s10008-020-04664-5

**Published:** 2020-05-29

**Authors:** Stanislav V. Sokolov, Richard G. Compton

**Affiliations:** 1 London, UK; 2 Department of Chemistry, Physical & Theoretical Chemistry Laboratory, Oxford University, South Parks Road, Oxford, OX1 3QZ UK

We thank Editor Fritz Scholz for the invitation to identify ‘Future Tasks of Electrochemistry’, which we are pleased to attempt from both the perspectives of academia (RGC) and industry (SVS). Before undertaking this, we wondered if such an exercise had been previously attempted and, if so, with what success? Accordingly, we considered the relatively unknown edition of the collected works of Alexander N Frumkin published in 1987 by the publisher Nauka under the editorship of Academic B.P. Nikolsky [1]. The two volumes are now out of print and found in just a few libraries, for example, Moscow State University. To the best knowledge of the authors, they have not been translated into the English language or available online.

Alexander Naumovich Frumkin (1895–1976), often thought of as the father of modern electrochemistry, was known for promoting electrochemistry and supervising future generations of scientists in addition to his major and transformative impact on the scientific development of the subject. He believed that electrochemistry would always play an important role in modern scientific developments and would cover multiple facets ranging from catalysis to the understanding of living cells. Frumkin’s thoughts on the future development of electrochemistry are best summarized in a plenary lecture he gave at the XI Mendeleev Meeting in 1975 at Alma-Ata Kazakhstan and which is summarized in the 1987 Nauka publication. It was Frumkin’s last plenary talk before his death in 1976 and was the culmination of multiple years of focused research. Since 1975, significant advances have been made yet a lot of the challenges highlighted remain as is briefly next discussed.

The original title of the talk was ‘On several problems in electrochemistry’. During the talk, Frumkin made a remark that a better title would be ‘On the utility of electrochemistry’ as the subject underpins energy storage and catalysis as well as industrial processes such as electroplating. ANF noted that the application of electrochemistry requires a full understanding of thermodynamics and kinetics. Furthermore, and in contrast to other branches of chemistry, (dynamic) electrochemical processes can be studied at or close to equilibrium conditions giving the example of exchange currents.

ANF asked, as we are again asked to question, what would the role of electrochemistry be in the future? He noted that a potential future of energy sources could be hydrogen based and that the understanding of electrolysis of water is required for large-scale hydrogen production. He imagined huge power stations splitting water and delivering hydrogen using pipelines to the cities.

ANF predicted in his lecture the development of electric cars, noting that batteries are fundamental components which determine the usability and range of a car. Frumkin noted that by apparently conservative estimates by the year 2000, electric cars would constitute 10% of all cars. Sadly that prediction has been missed with the records indicating the total percentage of electric vehicles at 2% in 2019 [2]. Development of modern electronics would also require appropriate energy sources capable of delivering increasing power from a small volume battery. Electrochemistry was, he emphasized, fundamental to understanding the physical processes behind power storage and offer rational ways forward for battery design.

ANF observed that at the time of writing approximately 12% of the annual worldwide production of metal was lost via corrosion processes. Reduction of corrosion would be increasingly important going forward with more complex alloys and structures being required for the development of the modern industry. It needed understanding of complex processes at liquid-solid-air interfaces and the generation of novel alloys capable of withstanding the harshest environments such as encountered during, for example, atomic energy production.

Two further interesting areas of future research were predicted: first, electro-synthesis given ANF’s recognition that solvated electrons can be electro-generated and might be used to drive subsequent organic reactions. Second, the need for novel quantum understanding of the double layer was required to explain the rate of reaction processes.

Towards the end of the lecture, Frumkin described his view of *the* key future problem of electrochemistry: bioelectrochemistry and understanding electrochemical processes in living systems. Frumkin used a quote by Faraday: ‘Wonderful as are the laws and phenomena of electricity when made evident to us in inorganic or dead matter, their interest can bear scarcely any comparison with that which attaches to the same force when connected with the nervous system and with life’ [3]. The generation of nerve impulse was given as an example of such process as was heart muscle contraction.

Many processes occur at the cellular membranes where the exchange of molecules takes place. ANF suggested that the then current studies with bilipid membranes were a starting point for more complex investigations of living systems and he expected that these complex processes could be simplified and understood using electrochemistry.

ANF reflected that electrochemistry is underappreciated science, often overshadowed by other aspects of chemistry and rarely mentioned in discussions of the future developments of chemistry. He concluded with the remarks: ‘My lecture might give an impression that I position electrochemistry in opposition to catalysis. That would be not correct. I fully understand the immense importance of heterogeneous catalysis, especially during our age dominated by the oil industry. But I just wanted to remind you that Aphrodite came from the sea foam, that is from a structured solution of electrolyte and not from oil.’ [4, 5].

The above lecture was presented in 1975. Frumkin’s vision was clearly very perceptive and whilst significant advances have been made in all the areas described, much of his agenda remains uncompleted perhaps most especially in the field of bioelectrochemistry in respect of coupling rigorous theoretical understanding with experimental data. The kinetics of enzymes remains an open area of focused interest especially in respect of understanding the behaviour at a single enzyme level [6, 7] particularly at a fundamental level. The ability to couple most electrodes to many enzymatic redox centres remains a challenge but which could potentially revolutionize the field of biosensors.

Of particular recent interest has been the development of so-called single-entity electrochemistry [8, 9] which allows the electrochemical study of not only single enzymes but also bacteria [10], blood cells [11] and even flu viruses [12]. We predict that these studies will provide an essential link between in vitro and in vivo studies allowing electrochemistry to break free from its almost exclusively molecular past and to embrace authentic complexity.

Frumkin said nothing about electrochemical sensors although electroanalysis was already highly developed at that time in the USSR not least through the work of the Tomsk group led by Armin Stromberg [13]. Arguably, this field became truly active worldwide with the invention in Oxford in the 1980s by H Allen O Hill of the blood glucose sensor [14, 15]. This massively transformed the quality of life for diabetics worldwide through allowing the self-analysis and monitoring of their blood glucose levels [16]. The future development of sensitive biosensors capable of detecting low concentrations of analytes in complex sample matrices will be the key to decentralized health care and to manage the spread of diseases such as COVID-19.

Electrochemistry still offers (but does not yet deliver) much novelty to the organic chemist interested in synthesis whilst promising green approaches to oxidation and reduction—at the heart of chemical bond making and breaking—through the use of ‘clean’ electrons (rather than using as now chemical oxidizing or reducing agents) and aqueous solutions. We predict this area will rapidly blossom over the coming few years not least since now there is currently a new generation of synthetic organic chemists who believe in the approach for addressing real synthetic challenges (as opposed to model studies as have frequently been studied in ‘physical organic chemistry’).

Generalizing beyond organic chemistry with its obvious potential impact in pharmaceutical design and production, we suggest the need to redesign the world’s chemical ‘heavy’ industry to make it green and renewable. This means in effect starting again and inventing processes from afresh recognizing the need for sustainability. This must be addressed holistically so that the entire manufacture of chemicals is changed from the bottom feedstocks up to the most sophisticated products of whatever scale. For certain, electrochemistry will lie at the heart of this new technology just as the electrolysis of brine to make sodium hydroxide and chlorine has traditionally underpinned much of the (heavy) chemical industry. The future generation of electricity will be from solar, hydroelectric, wind and tidal power sources with electrochemistry used to electrolyze water to make hydrogen plus oxygen and key to energy storage and transmission. The chemical starting materials will then be from abundant feedstocks such as water, carbon dioxide, nitrogen and biomass whilst designing products to allow the recycling of other elements of limited abundance. Whilst important work is currently active on the electrochemical activation of carbon dioxide, more is required on the even more challenging transformation of nitrogen into ammonia. Success will as pointed out by Frumkin in 1975 require a full and deep understanding of both thermodynamics. We should recall that probably the lowest point in the whole history of electrochemistry was the ‘cold fusion fiasco’ of 1989 [17] apparently based, in part, on a misunderstanding of the Nernst equation. This led to the observation of Martin Fleischmann, FRS and Stanley Pons that ‘It is inconceivable that this [amount of heat] could be due to anything but nuclear processes...’ We note that today’s expert electrochemists are not free from similarly basic errors as we have recently pointed out elsewhere [18].

Last, we note that electrochemistry has expanded in size, measured not least by the number of practitioners, vastly beyond any limits that Frumkin might have ever imagined. Moreover, it has changed qualitatively most importantly in respect of the diversity of its practitioners. The relative demise of the subject in the USA, the former USSR and the UK (especially since 2016 in the light of Brexit) has been more than offset by the revolutionary expansion in the PRC and to a lesser but still significant extent in Brazil and India. We expect further enrichment and scientific success via diversification, not least through the advancement of science in Africa, to have a continuing very positive transformative effect on the subject.
